# Transgenerational aspects of former Swiss child laborers: do second generations suffer from their parents’ adverse early-life experiences?

**DOI:** 10.3402/ejpt.v7.30804

**Published:** 2016-10-25

**Authors:** Andreas L. Küffer, Myriam V. Thoma, Andreas Maercker

**Affiliations:** 1 Division for Psychopathology and Clinical Intervention, Department of Psychology, University of Zurich, Zurich, Switzerland; 2 University Research Priority Program “Dynamics of Healthy Aging,” University of Zurich, Zurich, Switzerland; 3 Department of Psychiatry, University of California in San Francisco, San Francisco, CA, USA; 4 San Francisco Veterans Affairs Medical Center, San Francisco, CA, USA

**Keywords:** Childhood maltreatment, transgenerational effect, psychopathology, parental rearing behavior, sense of coherence, pessimism/optimism

## Abstract

**Background:**

Recent research suggests that childhood adversity exerts a lasting impact not only on the affected individuals but also on their offspring. Little is known about the role of parental rearing behavior in the transgenerational conveyance of parental childhood adversity and filial psychological health.

**Objective:**

Hence, it was the aim of the current study to investigate the relationship between parental rearing behavior of former Swiss indentured child laborers (“Verdingkinder”) and psychological health of their adult offspring.

**Methods:**

We applied a two-generation control-group design with two parental samples (*n*=16, former “Verdingkinder,” *M*_age_=76.13, *SD*=6.81 and *n*=19, parental controls, *M*_age_=72.63, *SD*=5.96) and their offspring (*n*=21, former “Verdingkinder” offspring, *M*_age_=52.91, *SD*=5.90, and *n*=29 offspring controls, *M*_age_=44.55, *SD*=7.71). Parental rearing behavior, childhood trauma, and psychological health were assessed with questionnaires. Data were analyzed using Bayesian analyses, where Bayes factors (BF) of 3 or higher were considered as substantial evidence for the tested hypotheses.

**Results:**

We found that “Verdingkinder” offspring reported more physical abuse (BF_10_=5.197) and higher total childhood trauma exposure (BF_10_=2.476). They described both their fathers (BF_10_=14.246) and mothers (BF_10_=24.153) as less emotional and their mothers as more punitive (BF_10_=18.725). An increased sense of reflection, for instance, one's ability to take different perspectives, was found in the offspring controls (BF_10_=5.245). Furthermore, exploratory analyses revealed that lower perceived familial emotionality was associated with higher psychopathology (all BF_10_=10.471) and higher pessimism (all BF_10_=5.396).

**Discussion:**

Our data provide cross-sectional evidence of a meaningful transgenerational relationship between parental childhood adversity, dysfunctional rearing behavior, and psychological health of offspring. Prospective studies are needed to investigate these findings in a longitudinal setting.

**Highlights of the article:**

- The study showed elevated levels of aversive childhood events in the adult offspring of former indentured childhood laborers.
- The offspring of former indentured childhood laborers did not exhibit increased general psychopathology (as assessed by the Symptom-Checklist: SCL).
- However, the recalled childhood rearing behavior was more problematic (higher punishment, less emotional warmth) in the offspring of former indentured childhood laborers as compared to a 2nd generation control group.
- Second generation group differences for world view variables (optimism, pessimism) were not substantial or in favor of the control participants (reflection facet of sense of coherence-revised).

Early-life adversity, such as trauma or maltreatment, can exert a “long shadow” (Brent & Silverstein, 2013), that is, a profound and, thus, long-lasting impact on the affected individual (Liu & Umberson, 2015; Springer, Sheridan, Kuo, & Carnes, 2003; Umberson, Williams, Powers, Liu, & Needham, 2005). The World Health Organization (WHO), which conducted a worldwide survey including over 50,000 respondents, concluded that childhood adversities are strongly associated with adult mental disorders and are found to predict 29.8% of DSM-IV disorders (Kessler et al., 2010). Stressful experiences in childhood are, thus, a strong predictor of adult mental health.

The impact of early-life adversity may go beyond the lifetime of the affected individual and spread to the next generation. In a study with Cambodian students, parental trauma symptomatology due to surviving the Khmer Rouge genocide as a child or adolescent was associated with anxiety and depression in offspring (Field, Om, Kim, & Vorn, 2011). In another study with Rwandan mothers who survived the genocide of 1994, it was found that maternal childhood violence exposure was associated with offspring anxiety, depression, and behavioral disorders (Roth, Neuner, & Elbert, 2014). Together, existing data derived from transgenerational research suggest that parentally experienced early-life adversity exerts a meaningful impact on the next generation's mental health.

Limited information exists with regard to the underlying mechanisms. One potential process responsible for the transgenerational conveyance of parentally experienced early-life adversity on offspring's mental health could be parenting style. On one hand, parenting style has repeatedly been found to be associated with offspring psychopathology, such as depression and anxiety (Lima, Mello, & Mari, 2010; Rapee, 1997), schizophrenia (Skagerlind, Perris, & Eisemann, 1996), personality disorders (Giakoumaki et al., 2013), and eating disorders (Tetley, Moghaddam, Dawson, & Rennoldson, 2014). On the other hand, parenting style has also been related to adverse childhood experiences. For instance, a group comparison between adult offspring of 159 Holocaust survivors and 151 controls revealed that Holocaust offspring experienced more parental punishment than controls (Kellermann, 2001). Similarly, in a Spanish sample with over 100 female hospital patients, it was found that perceived parental rearing style, that is, coldness, detachment, rejection, and parental overprotection, was related to self-reported childhood abuse and neglect (Hernandez et al., 2013). Similar results have been reported in a laboratory study of German mothers with self-reported early-life abuse and their 5-month-old infants. Mothers with a history of abuse were found to be more intrusive toward their infants as compared to mothers with no history of abuse (Moehler, Biringen, & Poustka, 2007). Taken together, existing data point toward a meaningful relationship between parental style and offspring's mental health as well as early-life adversity and parental rearing behavior.

Only a few studies exist that have looked at the mediating effect of parental rearing behavior on the relationship between parentally experienced early-life adversity and offspring mental health. In a series of investigations with Cambodian participants conducted by Field and colleagues, it was found that the association between parental trauma and offspring psychopathology was mediated by parenting style, that is, parent's role reversing and maternal overprotection (Field et al., 2011). However, conclusions are limited, as findings rely on self-reports of the teenage offspring only. In 2013, the authors conducted another two studies, this time including the Cambodian mothers, too. The authors were able to extend previous findings by showing that the parenting style of the mothers mediated the impact of maternal PTSD symptomatology on offspring's anxiety and depression (Field, Muong, & Sochanvimean, 2013). The authors were not only able to find the trauma transmission via parenting style in Cambodian dyads but also in Cambodian-American refugee dyads. Although these valuable studies point toward a meaningful moderator/mediator effect of parental rearing behavior on the relationship between parentally experienced childhood adversities and offspring's psychopathology, generalization of these results to parenting samples of Western origin involving more minor trauma is limited. Also, particular focus was put on mothers, restricting conclusions to mother–child pairs. Finally, hardly anything is known about the influence of parentally experienced early-life adversity on the offspring's resilience. A worthwhile exception is typified by a recently published study conducted with adult children of Holocaust survivors that included indicators of resilience, such as sense of coherence (SOC) (Fossion et al., 2014). In this study, it was shown that adult offspring from less functional families reported a lower SOC when compared to the general population, suggesting less resources to cope with stress (Fossion et al., 2014). However, it remains unclear whether this holds true only for the offspring of parents surviving genocide.

It was, therefore, the aim of the current study to examine the role of parental rearing behavior in the transgenerational transmission of parentally experienced early-adversity on psychological health of Verding-children's offspring. For this, we examined a historically unique sample of male and female former Swiss indentured child laborers, the so-called “Verdingkinder.” Until the late 1970s, it was a common practice in Switzerland to remove orphans, children of single-parent families, or even children from divorced or separated parents from their family environment and place them into indentured child labor. Children were mainly sent into farmers’ homes to work for their living. Historiographical studies have documented the harsh environment in which these individuals grew up, reporting that a large proportion of the children were regularly beaten, emotionally and sexually abused, and that some were even beaten to death (Furrer, Heiniger, Huonker, Jenzer, & Praz, 2014; Leuenberger & Seglias, 2008). Survivors of the former “Verdingkinder” are now in late life, and studies have reported high prevalence of adverse childhood experiences and poor mental health (Burri, Maercker, Krammer, & Simmen-Janevska, 2013; Maercker, Hilpert, & Burri, 2015; Maercker, Krammer, & Simmen-Janevska, 2014). Even though childhood trauma is not necessarily associated with former childhood labor, the sample of former “Verdingkinder” reported here was specifically selected for their childhood trauma in order to represent a common form of the “Verdingkind” phenomenon (Leuenberger & Seglias, 2008).

Our particular research questions were the following: Is there evidence for higher adverse childhood experiences in the offspring generation of parents that grew up at high risk of experiencing adversities and trauma compared to a control offspring generation? Is there evidence for dysfunctional parental rearing behavior in families of former “Verdingkinder”? Are there differences between the offspring samples with regard to psychopathology and psychological health? Furthermore, exploratory analyses investigated the interactions of parental rearing behavior and family type (“indentured child labor family” vs. “control family”) associated with offspring psychopathology and psychological health.

## Methods

### Sample and recruitment

Data for the parental sample of the former “Verdingkinder” have been collected within a larger study on long-term consequences of indentured child labor in Switzerland conducted by our research group between 2010 and 2012 (Burri et al., 2013; Kuhlman, Maercker, Bachem, Simmen, & Burri, 2013; Maercker et al., 2015). Within a project on biological consequences of childhood experiences and trauma, a subsample of former child laborers provided further information (Küffer et al., 2016). Data collection for the parental control sample was done within the context of the current study between 2013 and 2014. We expanded these samples with their offspring.

**Fig. 1:**
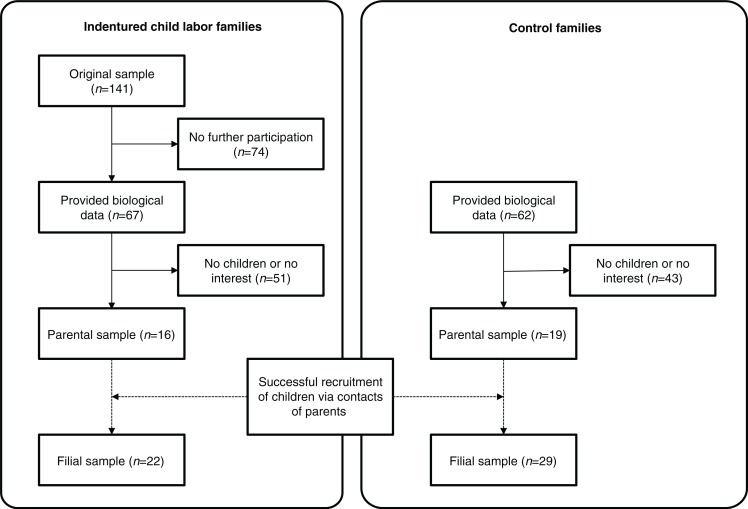
Flowchart of sample recruitment procedure.

Former indentured child laborers were recruited via advertisements in local and national newspapers and magazines and via particular associations and societies for former “Verdingkinder.” For the sample of former “Verdingkinder,” the following inclusion criteria were applied: (Swiss-)German speaking, a minimum age of 60 years, at least one period as an indentured child laborer, and a report of at least one traumatic event in their lifetime. The parental control sample was recruited via mouth-to-mouth advertising and mailing lists of the University of Zurich. Inclusion criteria included a minimum age of 60 years, (Swiss-)German speaking, upbringing in rural communities or in the countryside, having been raised by their biological parents, and being free of any psychiatric diagnosis, including PTSD. In order to be able to establish contact with the offspring of the parental samples, all participants with children were contacted in written form and asked to inform their children about the current study. Parental participants’ offspring were contacted by research coordinators via phone or email only if they showed interest in the study. All contacted children who expressed interest in participation were then provided with written study information material (see Fig. 1). Written informed consent was obtained from all the participants (parents and children), and the study procedure was approved by the ethical committee of the Canton of Zurich, Switzerland (KEK-ZH-Nr. 2012-0245).

### Procedure

Specifically, trained research assistants and graduate students interviewed the parental samples in one-to-one interviews. The interviews lasted between 2 and 3 h, including refreshment breaks, and were either conducted at the University of Zurich or at the participant's home, depending on the preference of the participants.

Data assessment of the offspring samples was conducted through paper and pencil questionnaires, which were sent to their homes by mail. A franked envelope was included with the questionnaire package. The completion of the questionnaire lasted approximately 30 min. Participants received no financial incentives. However, the parental samples were reimbursed for their travel expenses if the interview was held at the Psychological Institute of the University of Zurich.

### Instruments

The following instruments were used to assess parental and filial trauma exposure, recalled parental rearing behavior (from an offspring perspective), offspring psychopathology, offspring optimism/pessimism, and SOC.

#### Childhood Trauma Questionnaire–short form

The Childhood Trauma Questionnaire–short form (CTQ-SF) is a 28-item self-report inventory that provides brief and reliable screening for histories of abuse and neglect (Bernstein et al., 2003). It inquires about five types of maltreatment: emotional abuse, physical abuse, sexual abuse, emotional neglect, and physical neglect. The CTQ (including CTQ-SF) is one of the most widely used instruments to assess childhood maltreatment and trauma and has been extensively cross-validated (e.g., Karos, Niederstrasser, Abidi, Bernstein, & Bader, 2014). In the original validation study in a US community sample (*n*=1,007) aged between 18 and 65, Cronbach's *α* reached ranged from satisfying for the physical subscales (>58) to good in the case of emotional and sexual subscales (>83) (Scher, Stein, Asmundson, McCreary, & Forde, 2001).

#### Questionnaire of Recalled Parental Rearing Behavior

The questionnaire of recalled parental rearing behavior (QRPRB) is a 2×24-item German questionnaire for adult participants that assesses recalled maternal and paternal behavior in the dimensions of rejection and punishment, emotional warmth, control, and overprotection (Schumacher, Eisemann, & Brähler, 1999). Each subscale consists of eight items that are summed up. The QRPRB has been validated in a sample of *n*=2,968 participants and revealed internal consistency of Cronbach's *α*=0.72–0.89.

#### Brief Symptom Inventory

The Brief Symptom Inventory (BSI) consists of 53 items that assess nine psychological symptom dimensions: somatization, obsession-compulsion, interpersonal sensitivity, depression, anxiety, hostility, phobic anxiety, paranoid ideation, and psychoticism (Derogatis & Spencer, 1993; Thombs, Bernstein, Lobbestael & Arntz, 2009). For the current analysis, the global severity index (GSI) was used. The GSI is a sum score of all nine dimensions to indicate a participant's global, psychological distress. Cronbach's *α* for the nine subscales and the GSI was reported to be higher than 0.70 (Boulet & Boss, 1991).

#### Revised Life Orientation Test

Within the filial sample, optimism and pessimism were measured using the Revised Life Orientation Test (LOT-R) (Scheier, Carver, & Bridges, 1994). The instrument comprises three positively worded items, which comprise the optimism subscale (e.g., “In uncertain times, I usually expect the best”); three negatively worded items, which comprise the pessimism subscale (e.g., “If something can go wrong for me, it will”); and four filler items. Participants are asked to rate their agreement or disagreement with each of the statements on a five-point scale from 0 (“strongly disagree”) to 4 (“strongly agree”). Internal consistency for the optimism (Cronbach's *α*=0.88) and pessimism (Cronbach's *α*=0.78) subscales was high.

#### Sense of Coherence Revised

The SOC was assessed within the filial sample with Sense of Coherence Revised (SOC-R) (Bachem & Maercker, 2016). The questionnaire compromises 13 items that are divided into three dimensions of *manageability*, *reflection*, and *balance*. Items are assessed on a five-point scale from 0 (“not at all”) to 4 (“completely”). In a sample of 60 bereaved persons, the SOC-R had a test–retest reliability of 0.85 over the period of 4 weeks and one of 0.75 for the period of 15 months. In the validation study of Bachem and Maercker (2016) that analyzed two different samples, the Cronbach's *α* ranged from 0.57 (for balance) to 0.77 (for manageability). The total scale had Cronbach's *α* of 0.75 and 0.81.

#### Covariates

Additional study covariates were age, sex, self-evaluated financial situation (from “poor” to “very good”), and total years of education. Self-evaluated financial situation (i.e., “How would you describe your financial situation right now?”) was used as a proxy for socio-economic status.

### Statistical analysis

Data handling was conducted with R (R core Team, 2013), and statistical analysis was conducted with R and JASP, an open source software for Bayesian data analyses (Love et al., 2015). Because some participants from the filial generation were siblings, the data presented here were nested within the 35 families, and therefore, theoretically non-independent. Therefore, intraclass correlation-coefficient (ICC) (Bliese, 2000) was assessed for all potential dependent variables. Because all ICC were trivial (all ICC<0.10), all analyses were conducted under the assumption of statistical independence.

Because of the relatively small sample sizes and the fact that not all dependent variables were normally distributed, Bayes factors (BF) were computed with Bayesian *t*-tests and Bayesian analyses of covariance (BANCOVA) for hypotheses testing and exploratory analyses of interactions, instead of conventional inference by null-hypotheses testing (Rouder, Morey, Speckman, & Province, 2012; Rouder, Speckman, Sun, Morey, & Iverson, 2009).

According to Van de Schoot et al. (2014), in Bayesian statistics the key difference to null-hypotheses significance testing (NHST) lies in the nature of the unknown parameters. The framework of NHST assumes that the parameters of interest are unknown, but fixed in the true population. For example, in the context of our study, one true CTQ mean could be determined for the whole population of all former indentured child laborers. The Bayesian standpoint assumes that unknown parameters are uncertain and should therefore be described by probability distributions. For example, the mean of the CTQ score in the population of former indentured child laborers is not fixed and rather expressed in the form of a probability distribution. In order to estimate this probability distribution, Bayesian inference relies on three “ingredients” (Van de Schoot et al., 2014). The first concerns the background knowledge on the parameters, a researcher gathers before model testing. This background knowledge is reflected in the *prior distribution*, which then comes to represent the assumptions of a researcher before analyzing the data. Furthermore, the variance of the prior distribution describes the level of uncertainty about an investigated population value. For instance, if the researcher expects no specific effect between two populations and assumes that every effect is possible but more probable for small effects (either positive or negative), the prior distribution variance is large. In this example, there is only little certainty about the population value of the expected effect. Given more information, though, the researcher can restrict the variance of the prior distribution by using a more clearly defined effect. As an example from our study, if there was no reason to assume a specific effect between the control sample and the “Verdingkinder” sample, we did not restrict the prior distribution, while assuming that smaller effects were more probable than larger effects. On the other hand, if we had reason to expect a specific directionality for the effect, we restricted the variance of the effect in the given direction. For instance, we expected lower total CTQ scores for controls versus former indentured child laborers. Therefore, the variance of the effect was not allowed to be negative for this analysis. Despite an increased precision for the second case with restricted variance for the expected effect, both prior distributions are fairly low in precision and are therefore referred to as low-informative prior distributions. The important step in Bayesian approaches is the distribution of the assessed data that is expressed in terms of the *likelihood function* (Van de Schoot et al., 2014). Via Bayes’ theorem, the prior distribution and the likelihood function of the assessed data are reconciled into the *posterior distribution*. The posterior distribution reflects the updated knowledge of our investigated parameters and consists of prior knowledge, weighted by the evidence gathered in a given study.

One way to draw statistical inference with Bayesian approaches is the use of BFs (Andraszewicz et al., 2015). BFs grade the decisiveness of the evidence by comparing the posterior beliefs for H_0_ against the posterior beliefs for H_1_. With other words, a BF can be expressed as odds for support or evidence for the H_0_ over H_1_ (BF_01_) or as support for H_1_ over H_0_ (BF_10_). A practical guide on Bayesian approaches by Jarosz and Wiley (2014) lists verbal labels that allow a more intuitive interpretation of these BFs/odds. For instance, Wetzels et al. (2011) interpret a BFs_10_<1 as non-evidential, BFs from 1 to 3 as anecdotal or weak, BFs from 3 to 10 as substantial evidence for the H_1_, BFs from 10 to 30 as strong evidence, BFs from 30 to 100 as very strong evidence, and BFs>100 as decisive evidence. It is important to understand that BFs represent an odds-ratio in favor or against tested hypotheses or models, and those listed labels can be interpreted on a continuum, rather than on the basis of discreet thresholds (as for instance with significance levels of *p*-values).

Furthermore, Bayesian approaches have several distinctive advantages over NHST (Van de Schoot et al., 2014). Firstly, with the use of BFs it is possible to compare specific models and hypotheses directly (Andraszewicz et al., 2015), whereas NHST classically informs about the probability of some data, given the H_0_ (Cohen, 1994). Therefore, Bayesian inference with the use of BFs allows for an interpretation of the evidence against any given hypothesis, thus, also against the H_0_. NHST on the other hand only permits us to reject the H_0_ if a *p*-value falls below the significance level, but does not facilitate an interpretation of the result in favor of the H_0_ in case of a non-significant *p*-value. Furthermore, an advantage of Bayesian statistics over NHST is that Bayesian approaches work with the given data directly, rather than assuming that they behave like data that are normally distributed. In the case of non-normal distributed parameters, Bayesian analyses provide more accurate results and allow statistical inference even in the absence of normality (Van de Schoot et al., 2014). Bayesian approaches are also well-suited to deal with small sample sizes. Even though the posterior distribution is more affected by the choice of the prior distribution if the sample size is smaller (due to fewer evidence) (Van de Schoot et al., 2014), Bayesian inference remains a better choice for small sample sizes than NHST. This seems to be also the case if default prior settings were chosen, as it was the case in our analyses.

See Van de Schoot et al. (2014) and/or Andraszewicz et al. (2015) for a more detailed introduction to Bayesian inference.

JASP allows us to specify the direction of group difference one expects in group comparisons, by restricting the prior distribution of the effect size—in this case, a default Cauchy prior=0.707—to be either positive or negative. Therefore, if our hypotheses suggested a specific direction for group differences (e.g., more adverse childhood experiences in children from former indentured child laborers compared to children from a parental control group), Bayesian *t*-tests were tested accordingly.

For the BANCOVAs, the BF_10_ indicates the evidence for the full models (groups×parental rearing behavior variable) over the null model. Supplementary analyses (see Supplementary Table 3) did not indicate that perceived rearing behavior from participating partners (i.e., parents that actively participated as former indentured child laborers or controls) differed from absent partners (i.e., parents we did not have data on, except the perceived rearing behavior scores from the respective offspring). Consequently, we aggregated maternal and paternal rearing behavior scores into family level variables by adding up scores of parental dyads. This was done under the assumption that one aspect of parental rearing behavior (i.e., maternal or paternal) cannot affect offspring psychopathology and well-being without interacting with the complementary aspect. Given that gender, financial status, and age of the participants were controlled for, these variables were included in the null model.

Our assumption was that children who described more family punishment, less emotionality, and higher control indicated more psychopathology, a worse SOC, and less optimism/more pessimism. Furthermore, we mainly expected this pattern in families from former indentured child laborers. Therefore, we planned to analyze our date with nine separate analyses of covariance.

## Results

### Sample characteristics

In total, *n*=35 participants from the parental generation, including *n*=16 former “Verdingkinder” and *n*=19 parental controls with no history of childhood maltreatment, and *n*=51 participants from the filial generation, including *n*=22 adult children from former “Verdingkinder” and *n*=29 adult children from parental controls, were enrolled in this study. The mean age of both parental samples was *M*=74.31 (*SD*=6.83) with *n*=16 women (40.0%). The mean age of both filial samples was *M*=48.16 (*SD*=8.09) with *n*=35 women (68.7%). Table 1 shows the sample characteristics. Bayesian frequency analyses suggested that there was no evidence for differences in gender, marital status, or financial status in the parental samples. Although we found no real support for an age difference, we found a strong difference in years of education in the parental samples (BF_10_=31.350). Regarding the filial samples, the frequency analyses supported no gender difference and no difference in marital status or financial status. Regarding age, the difference was very strong (BF_10_=214.87), but there were no differences in years of education.

**Table 1:** Sample characteristics of parental and filial data, divided by groups

			ICL		Controls		
					
			*M (SD)*	*n* (%)	*M (SD)*	*n* (%)	BF_10_
Parental generation	*N*		16		19		
	Females		6	(37.50)	8	(42.11)	0.589
	Age		76.13	(6.81)	72.63	(5.96)	1.291
	Education		10.71	(1.65)	13.50	(2.99)	31.350
	Marital status	Married	8	(50.00)	11	(57.89)	0.618
		Separated	1	(6.25)	3	(15.79)	
		Widowed	7	(43.75)	5	(26.32)	
	Financial status	Poor	1	(6.25)	0	(0.00)	0.785
		Fair or good	13	(81.25)	11	(57.90)	
		Very good	2	(12.50)	8	(42.11)	
Filial generation	*N*		22		29		
	Females		15	(68.18)	20	(68.97)	0.458
	Age		52.91	(5.90)	44.55	(7.71)	>100
	Education		13.91	(3.77)	15.55	(3.07)	0.933
	Marital status	Single	3	(13.64)	9	(31.03)	0.531
		Married	12	(54.55)	15	(51.72)	
		Separated	7	(31.82)	5	(17.24)	
	Financial status	Poor	2	(9.09)	0	(0.00)	1.089
		Fair or good	14	(64.64)	23	(79.31)	
		Very good	6	(27.27)	6	(20.69)	

Independent sample *t*-tests and contingency tables were validated with a Bayesian approach (instead of null hypotheses significance testing). The here presented analyses were non-directional hypotheses in order to check if the two samples did not differ in demographic measures. ICL=Indentured child labor group.

### Early-life adversity and trauma across generations

In a first step, we tested whether the parental samples differed with regard to early-life adversity. We found that the CTQ-SF group differences were strong for all dimensions (see Table 2), indicating meaningful differences in early-life adversity and trauma between parental samples. In a next step, we compared scores of the various subscales of the CTQ-SF in the offspring samples. Consistent with the parental findings, the offspring “Verdingkind” sample reported higher childhood trauma exposure than the offspring control sample (see Table 2). There were substantial differences between self-reported total CTQ-SF (BF_10_=2.476) and physical abuse (BF_10_=5.197), whereas emotional abuse (BF_10_=1.812) and emotional neglect (BF_10_=1.594) were only anecdotal, and those of physical neglect and sexual abuse yielded no support for our hypothesis (both BFs_10_<1).

**Table 2:** Group differences on CTQ-SF scores across generations

		ICL		Controls		
				
		*M*	*SD*	*M*	*SD*	BF_10_
		Parental generation				
CTQ-SF	Total	70.95	22.18	35.47	7.25	>100
	Emotional abuse	12.61	6.72	6.63	2.31	66.21
	Physical abuse	13.31	7.07	6.16	2.27	>100
	Sexual abuse	10.30	6.94	5.42	1.02	17.96
	Emotional neglect	20.00	4.72	9.90	3.68	>100
	Physical neglect	14.38	4.54	7.37	2.36	>100
		Filial generation				
CTQ-SF	Total	40.23	16.64	33.34	8.21	2.476
	Emotional abuse	8.82	3.09	6.83	5.04	1.812
	Physical abuse	6.77	3.78	5.10	0.41	5.197
	Sexual abuse	5.86	2.21	5.62	1.93	0.392
	Emotional neglect	11.73	5.85	9.45	3.99	1.594
	Physical neglect	7.05	3.05	6.34	1.84	0.709

Independent sample *t*-tests were validated with a Bayesian approach (instead of null hypotheses significance testing). It was assumed that indentured child labor (ICL) families indicated more childhood adversities and therefore, tested in a directional manner. CTQ-SF=childhood trauma questionnaire.

### Parental rearing behavior, psychopathology, and resilience in offspring samples

In a next step, we analyzed whether the offspring samples differed with regard to self-reported, recalled parental rearing behavior, psychopathology, optimism/pessimism, and SOC. For the parental rearing behavior, scores were computed separately for maternal and paternal behavior. In line with our hypotheses, albeit only weakly for the paternal side, parental punishment has been found to be higher in the “Verdingkind” families (see Table 3). The “Verdingkind” offspring sample reported distinctly less emotionality by their fathers as compared to the control offspring sample (BF_10_=14.246). No difference was found in recalled paternal control and overprotection (both BFs_10_<1.231). Consistent with the paternal findings—but more pronounced—the same differences were found in maternal recalled rearing behavior. Controls reported to have perceived more emotionality by their mothers (BF_10_=23.153) and less punishment (BF_10_=18.724). As in the paternal control sample, there was no evident difference in maternal control between the two samples (BF_10_=0.826; see Table 3).

**Table 3:** Group differences between the two filial samples on the QRPRB, the BSI, the SOC-R and the LOT-R

		ICL		Controls		
				
		*M*	*SD*	*M*	*SD*	BF_10_
QRPRB						
Father	Punishment	11.27	4.13	9.97	2.13	1.231
	Emotionality	17.73	6.19	22.26	5.09	14.246
	Control	12.52	3.19	12.91	2.50	0.418
Mother	Punishment	12.41	4.32	9.67	1.74	23.153
	Emotionality	20.05	3.92	23.93	5.36	18.725
	Control	14.50	4.26	13.34	2.89	0.826
BSI	GSI	18.41	17.31	15.97	19.83	0.408
SOC-R	Total	38.42	6.01	41.62	5.70	2.435
	Manageability	15.68	3.34	16.10	2.58	0.479
	Reflection	12.19	2.28	13.66	2.11	5.245
	Balance	10.55	3.31	11.86	2.74	1.386
LOT-R	Total	20.05	4.91	21.17	4.46	0.596
	Pessimism	4.32	2.78	3.03	2.54	1.741
	Optimism	9.21	2.68	9.36	2.70	0.246

Independent sample *t*-tests were validated with a Bayesian approach (instead of null hypotheses significance testing). Indentured child labor (ICL) offspring were assumed to report more parental punishment, less emotionality and more control, more psychopathology and a lower sense of coherence. Furthermore, they were assumed to indicate more pessimism and less optimism. Hence, hypotheses were tested unidirectional. BSI-GSI=brief symptom inventory global severity index; QRPRB=questionnaire of recalled parental rearing behavior; SOC-R=sense of coherence revised questionnaire; LOT-R=revised life orientation test.

We found no meaningful difference in psychopathology, as measured with the BSI-GSI (BF_10_=0.408), indicating no difference in mental health between offspring samples. Also, we found no evidence for a difference in optimism. However, “Verdingkinder” offspring reported anecdotally more pessimistic views than their controls (BF_10_=1.741). Finally, with regard to SOC, the total score of the SOC-R revealed an anecdotal difference between groups (BF_10_=2.560), indicating a stronger SOC in the offspring control sample. This difference seemed to be mostly driven by the subscale “reflection,” as the higher scores of the offspring control sample were substantially different (BF_10_=5.245), whereas the difference in the “balance” subscale was anecdotal (BF_10_=1.386), and the difference in “manageability” was irrelevant in regard to our hypothesis (BF_10_=0.399; see Table 3).

### Associations between parental rearing behavior, family type, and mental health of the offspring generation

With this step, we tested our exploratory hypothesis that parental rearing behavior depends on family type. As indicated above, maternal and paternal rearing behaviors for each participant were aggregated into three family level variables: family punishment, family emotionality, and family control. To analyze whether family/group affiliation interacted with parental rearing behavior, a 2 (“indentured child labor family” vs. “control family”)×1 (“parents rearing behavior”) BANCOVAs was conducted. The GSI score, SOC-R total, and the LOT-R pessimism score were outcome variables in this analysis. All models were subsequently controlled for offspring's age and gender. QRPRB scores of paternal and maternal control did not differ between groups and were, therefore, dropped for these analyses. This resulted in a total of six BANCOVAs (see Table 4).

**Table 4:** Outputs of group×family emotionality and group×family punishment Bayesian analyses of covariance

Group×family emotionality			
Outcome variables	BSI-GSI	SOC-R	Pessimism
Group	0.647	2.398	0.377
Family emotionality	16.839	0.393	23.151
Group×family emotionality	1.757	0.347	0.466
**BF**_**10**_	**10.471**	**0.623**	**5.356**
Group×family punishment			
Outcome variables	BSI-GSI	SOC-R	Pessimism
Group	0.26	3.22	0.365
Family punishment	0.704	0.441	0.772
Group×family emotionality	0.207	1.01	0.392
**BF**_**10**_	**0.133**	**1.636**	**0.278**

Analyses of covariance were validated with a Bayesian approach (instead of null hypotheses significance testing). Bayes factors (BF_10_: in bold) indicate the evidence for the full models (groups×parental rearing behavior variable) over the null model. Since gender, financial status, and age of the participants were controlled for, these variables were included in the null model. Non-bold BF_10_ indicate analyses of effects within the tested model.

**Fig. 2:**
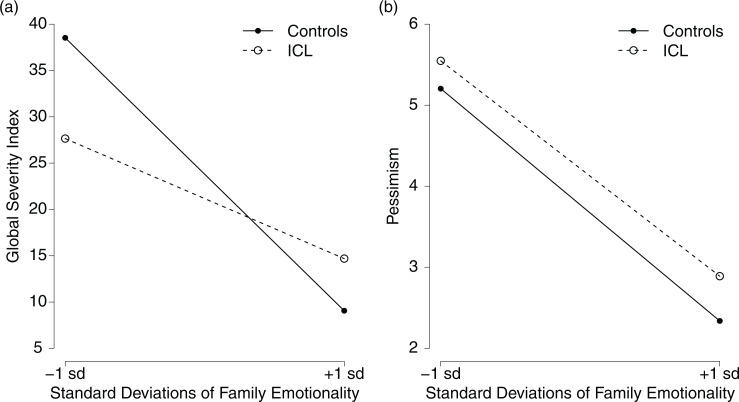
Interaction plots for family emotionality and group affiliation on outcome variables. Family emotionality is represented with±*SD* from the mean. (a) Outcome variable is offspring psychopathology, as indexed by the brief symptom inventory global severity index. (b) Outcome variable is offspring pessimism, measured by the pessimism scale of the revised life orientation test.

The models analyzing family emotionality were tested for main effects of group affiliation and family emotionality, as well as their interactions. The models revealed strong evidence for psychopathology (BF_10_=10.471), and substantial evidence for pessimism (BF_10_=5.356), indicating that psychopathology and pessimism are both affected by family emotionality and group affiliation. As can be seen in Fig. 2, both of these results were determined by the main effect of family emotionality on psychopathology and family emotionality on pessimism. Participants with lower family emotionality reported more psychopathology and pessimism. The main effect of group was insignificant in both models (both BF_10_<0.647). The interaction term of group×family emotionality appeared only anecdotal in the case of psychopathology (BF_10_=1.757) and was not evident in pessimism (BF_10_=0.466). The association with SOC appeared to be non-evidential (BF_10_=0.623), indicating that psychopathology might be partially dependent on family affiliation, whereas pessimism and SOC remained independent from it.

When focusing on family punishment and group affiliation, the BANCOVAs revealed only anecdotal evidence for an association with SOC (BF_10_=1.636), but no evidence for associations with psychopathology or pessimism (both BFs_10_<0.278), indicating that family punishment and group affiliation did not affect psychological health of offspring in an evident manner.

## Discussion

It was the aim of the current study to investigate the relationship between parental rearing behavior of former Swiss indentured child laborers, so-called “Verdingkinder” and psychological health of their adult offspring. We found that former “Verdingkinder” reported more adverse childhood experiences than controls. A similar pattern was observed in the “Verdingkinder” offspring sample, yet not as distinct as for the parental sample. What is more, although we found no meaningful group difference with regard to offspring's psychopathology, the “Verdingkinder” offspring sample reported substantially more physical abuse, and somewhat more emotional abuse and emotional neglect than their controls. Furthermore, the “Verdingkinder” offspring sample described their mothers and fathers as less emotional and described their mothers—and to a lesser extent their fathers—as more punitive. Although we found no differences in offspring's psychopathology, controls described themselves with a stronger SOC, mainly because of more reflection and a less pessimistic view. Further exploratory analyses indicated that lower family emotionality was associated with more psychopathology and more pessimism, albeit mostly independent from group affiliation.

Our finding of higher levels of adversities in the parental sample of “Verdingkinder” was expected and in line with previous findings of our research group (Küffer et al., in press). We did not find elevated levels of psychopathology in the “Verdingkinder” offspring sample when compared to offspring controls. This finding is consistent with results of a meta-analytic investigation studying secondary traumatization in the offspring of Holocaust survivors (Van IJzendoorn, Bakermans-Kranenburg, & Sagi-Schwartz, 2003), where the authors found no evidence for a transgenerational conveyance of parental trauma. Nonetheless, although levels of psychopathology were not elevated, the “Verdingkinder” offspring sample reported more childhood adversities and trauma, indicating that the number of experienced adversities and trauma but not levels of psychopathology are higher in the offspring of trauma survivors.

Similar to the findings by Roth et al. (2014), our data do not support the notion of a “simple” transgenerational trauma transmission. In Roth et al. (2014), the authors did not find a direct association between maternal trauma or maternal PTSD and offspring psychopathology, and instead identified maternally experienced childhood violence (i.e., the violence, the mother experienced during her childhood) as an important factor related to offspring psychopathology. The authors suggested that the familial context, such as maternal rearing behavior or parenting capacities, might have acted as a transmitting mechanism of maternally experienced early-life adverse experiences. Likewise, our results point in this direction, as in our sample, parental rearing behavior of former “Verdingkinder” has been described as more harmful by their offspring as compared to parental rearing behavior of controls. Unlike Field et al. (2011, 2013), who found that role-reversing parenting and parental overprotection were related to offspring's psychopathology (i.e., anxiety), we found that mainly maternal punishment and parental emotionality are the key aspects of “Verdingkind” parental rearing behavior (while we did not assess role-reversing in our study). These differing findings may be explained by the fact that analyses by Field and colleagues were within-group analyses of Cambodian Khmer Rouge survivor offspring, whereas we compared two different Swiss samples. It is, therefore, possible that within trauma survivors, overprotection is the most important aspect of parental rearing behavior, but when focusing on differences between groups, punishment and emotionality are more relevant. Although one expects within-group variance in a given population, one does not necessarily expect differences in this dimension in our two samples, because the two groups shared most of their cultural frame of reference. The dimension of control and overprotection might be context- and culture-specific and might be shared in the cultural context of our investigated groups.

We also found a weaker SOC in the offspring of “Verdingkinder,” which is in line with findings reported by Fossion et al. (2014), who found lower levels of resilience in adult offspring of less functional families. It is important to note here that we used a revised version of the original SOC questionnaire (i.e., SOC-R; Bachem & Maercker, 2016), whereas Fossion et al. (2014) applied the original construct by Antonovsky (1987). According to Bachem and Maercker, the SOC-R holds more distinctive variance, a better factor structure, and more stability over Antonovsky's (1987) SOC. Our finding adds, thus, to the growing body of literature that offspring of trauma survivors suffer not necessarily because of elevated psychopathology, but because of weakened resiliency capacities. But then again, one has to argue that although “Verdingkinder” offspring did experience more adversities and trauma, and experienced less than optimal parental rearing behavior, they did not develop elevated levels of psychopathology (as could have been expected), which might be considered a resilient aspect.

This study has a number of limitations: The sample sizes of both the parental and the offspring samples were rather small. Because of that, group comparisons allowed only for detection of larger effects, and most of the reported results—despite a trend in the assumed direction—remained anecdotal, or revealed to be statistically insignificant. In addition, our study was correlational, not allowing us to make causal conclusions. Therefore, our results should be replicated in a bigger sample, applying a prospective study design. Also, the selection of former child laborer families might not be representative with regard to family functionality. For ethical reasons, participants of the parental generation were asked to establish contact with their children by themselves and not by the research assistants. It is, therefore, possible and also confirmed by some of the former indentured child laborers that the relationship with their children was dysfunctional to the extent that they broke contact with their children altogether. As a consequence, our offspring sample might be a positive selection, implying that a more representative sample would have yielded more pronounced effects. Finally, our samples were not completely matched with regard to sample characteristics. The parental samples differed with regard to age and also reported substantially different levels of education. Although the age difference may be considered rather small, and the difference in years of education was fairly large. This can partially be explained by the fact that many former “Verdingkinder” were not given full access to education as compared to children with no “Verdingkind” background (Leuenberger & Seglias, 2008). It follows that a comparatively low level of education might be part of the demographic profile as a former child laborer. Future studies should match their samples with regard to these variables to exclude the possibility that our results were confounded by differences in demographic characteristics. The filial samples also meaningfully differed with regard to age. It might be speculated that, as a consequence of a lower level of education, former child laborers started their family earlier (Rindfuss, Morgan, & Offutt, 1996).

In conclusion, this is the first study that explored the transgenerational consequences of adverse childhood experiences in a sample of elderly former Swiss childhood laborers and their offspring. Future studies should include additional constructs from the positive spectrum of psychological health and functioning in order to approximate resilience more accurately.

## References

[CIT0001] Andraszewicz S, Scheibehenne B, Rieskamp J, Grasman R, Verhagen J, Wagenmakers E.-J (2015). An introduction to Bayesian hypothesis testing for management research. Journal of Management.

[CIT0002] Antonovsky A (1987). Unraveling the mystery of health: How people manage stress and stay well.

[CIT0003] Bachem R, Maercker A (2016). Development and psychometric evaluation of a revised sense of coherence scale. European Journal of Psychological Assessment.

[CIT0004] Bernstein D.P, Stein J.A, Newcomb M.D, Walker E, Pogge D, Ahluvalia T, Zule W, … (2003). Development and validation of a brief screening version of the Childhood Trauma Questionnaire. Child Abuse & Neglect.

[CIT0005] Bliese P.D, Klein K.J, Kozlowski S.W.J (2000). Within-group agreement, non-independence, and reliability: Implications for data aggregation and analysis. Mulitylevel theory, research, and methods in organizations.

[CIT0006] Boulet J, Boss M.W (1991). Reliability and validity of the Brief Symptom Inventory. Psychological Assessment.

[CIT0007] Brent D.A, Silverstein M (2013). Shedding light on the long shadow of childhood adversity. The Journal of the American Medical Association.

[CIT0008] Burri A, Maercker A, Krammer S, Simmen-Janevska K (2013). Childhood trauma and PTSD symptoms increase the risk of cognitive impairment in a sample of former indentured child laborers in old age. PLoS One.

[CIT0009] Cohen J (1994). The earth is round (p<05). American Psychologist.

[CIT0011] Derogatis L.R, Spencer P (1993). Brief symptom inventory: BSI.

[CIT0012] Field N.P, Muong S, Sochanvimean V (2013). Parental styles in the intergenerational transmission of trauma stemming from the Khmer Rouge regime in Cambodia. American Journal of Orthopsychiatry.

[CIT0013] Field N.P, Om C, Kim T, Vorn S (2011). Parental styles in second generation effects of genocide stemming from the Khmer Rouge regime in Cambodia. Attachment & Human Development.

[CIT0014] Fossion P, Leys C, Vandeleur C, Kempenaers C, Braun S, Verbanck P, Linkowski P (2014). Transgenerational transmission of trauma in families of Holocaust survivors: The consequences of extreme family functioning on resilience, sense of coherence, anxiety and depression. Journal of Affective Disorders.

[CIT0015] Furrer M, Heiniger K, Huonker T, Jenzer S, Praz A.-F (2014). Fürsorge und Zwang: Fremdplatzierung von Kindern und Jugendlichen in der Schweiz 1850–1980. Entre assistance et contrainte: le placement des enfants et des jeunes en Suisse 1850–1980.

[CIT0016] Giakoumaki S.G, Roussos P, Zouraraki C, Spanoudakis E, Mavrikaki M, Tsapakis E.M, Bitsios P (2013). Sub-optimal parenting is associated with schizotypic and anxiety personality traits in adulthood. European Psychiatry.

[CIT0017] Hernandez A, Gallardo-Pujol D, Pereda N, Arntz A, Bernstein D.P, Gaviria A.M, Gutierrez-Zotes J.A, … (2013). Initial validation of the Spanish Childhood Trauma Questionnaire-short form: Factor structure, reliability and association with parenting. Journal of Interpersonal Violence.

[CIT0018] Jarosz A, Wiley J (2014). What are the odds? A practical guide to computing and reporting Bayes factors. The Journal of Problem Solving.

[CIT0020] Karos K, Niederstrasser N, Abidi L, Bernstein D.P, Bader K (2014). Factor structure, reliability, and known groups validity of the German version of the Childhood Trauma Questionnaire (Short-form) in Swiss patients and nonpatients. Journal of Child Sexual Abuse.

[CIT0021] Kellermann N.P.F (2001). Perceived parental rearing behavior in children of Holocaust survivors. Israel Journal of Psychiatry and Related Sciences.

[CIT0022] Kessler R.C, McLaughlin K.A, Green J.G, Gruber M.J, Sampson N.A, Zaslavsky A.M, Williams D.R, … (2010). Childhood adversities and adult psychopathology in the WHO world mental health surveys. British Journal of Psychiatry.

[CIT0047] Küffer A, O’Donovan A, Burri A, Maercker A (2016). Posttraumatic stress disorder, adverse childhood events, and buccal cell telomere length in elderly swiss former indentured child laborers. Frontiers in Psychiatry.

[CIT0023] Kuhlman K.R, Maercker A, Bachem R, Simmen K, Burri A (2013). Developmental and contextual factors in the role of severe childhood trauma in geriatric depression: The sample case of former indentured child laborers. Child Abuse & Neglect.

[CIT0024] Leuenberger M, Seglias L (2008). Versorgt und vergessen: ehemalige Verdingkinder erzählen.

[CIT0025] Lima A.R, Mello M.F, Mari J.D.J (2010). The role of early parental bonding in the development of psychiatric symptoms in adulthood. Current Opinion in Psychiatry.

[CIT0026] Liu H, Umberson D (2015). Gender, stress in childhood and adulthood, and trajectories of change in body mass. Social Science & Medicine.

[CIT0027] Love J, Selker R, Marsman M, Jamil T, Verhagen A.J, … Ly A (2015). JASP. http://jasp-stats.org/download/.

[CIT0028] Maercker A, Hilpert P, Burri A (2015). Childhood trauma and resilience in old age: Applying a context model of resilience to a sample of former indentured child laborers. Aging & Mental Health.

[CIT0029] Maercker A, Krammer S, Simmen-Janevska K, Markus F, Kevin H, Thomas H, Sabine J, Anne-Françoise P (2014). Psychische Folgestörungen der Verdingung im Alter. Fürsorge Und Zwang: Fremdplatzierung von Kinder Und Jugendlichen in Der Schweiz 1850–1980.

[CIT0030] Moehler E, Biringen Z, Poustka L (2007). Emotional availability in a sample of mothers with a history of abuse. The American Journal of Orthopsychiatry.

[CIT0048] R Core Team (2013). R: A language and environment for statistical computing. https://cran.rproject.org/doc/FAQ/R-FAQ.html.

[CIT0031] Rapee R (1997). Potential role of childrearing practices in the development of anxiety and depression. Clinical Psychology Review.

[CIT0032] Rindfuss R.R, Morgan S.P, Offutt K (1996). Education and the changing age pattern of American fertility: 1963–1989. Demography.

[CIT0033] Roth M, Neuner F, Elbert T (2014). Transgenerational consequences of PTSD: Risk factors for the mental health of children whose mothers have been exposed to the Rwandan genocide. International Journal of Mental Health Systems.

[CIT0034] Rouder J.N, Morey R.D, Speckman P.L, Province J.M (2012). Default Bayes factors for ANOVA designs. Journal of Mathematical Psychology.

[CIT0035] Rouder J.N, Speckman P.L, Sun D, Morey R.D, Iverson G (2009). Bayesian t tests for accepting and rejecting the null hypothesis. Psychonomic Bulletin & Review.

[CIT0036] Scheier M.F, Carver C.S, Bridges M.W (1994). Distinguishing optimism from neuroticism (and trait anxiety, self-mastery, and self-esteem): A reevaluation of the life orientation test. Journal of Personality and Social Psychology.

[CIT0037] Scher C.D, Stein M.B, Asmundson G.J.G, McCreary D.R, Forde D.R (2001). The childhood trauma questionnaire in a community sample: Psychometric properties and normative data. Journal of Traumatic Stress.

[CIT0038] Schumacher J, Eisemann M, Brähler E (1999). Rückblick auf die Eltern: Der Fragebogen zum erinnerten elterlichen Erziehungsverhalten (FEE). Diagnostica.

[CIT0039] Skagerlind L, Perris C, Eisemann M (1996). Perceived parental rearing behaviour in patients with a schizophrenic disorder and its relationship to aspects of the course of the illness. Acta Psychiatrica Scandinavica.

[CIT0040] Springer K.W, Sheridan J, Kuo D, Carnes M (2003). The long-term health outcomes of childhood abuse: An overview and a call to action. Journal of General Internal Medicine.

[CIT0041] Tetley A, Moghaddam N.G, Dawson D.L, Rennoldson M (2014). Parental bonding and eating disorders: A systematic review. Eating Behaviors.

[CIT0042] Thombs B.D, Bernstein D.P, Lobbestael J, Arntz A (2009). A validation study of the Dutch Childhood Traum Questionnaire-Short Form: Factor structure, reliability, and known-groups validity. Child Abuse & Neglect.

[CIT0043] Umberson D, Williams K, Powers D.A, Liu H, Needham B (2005). Stress in childhood and adulthood: Effects on marital quality over time. Journal of Marriage and Family.

[CIT0044] Van de Schoot R, Kaplan D, Denissen J, Asendorpf J.B, Neyer F.J, Van Aken M.A (2014). A gentle introduction to Bayesian analysis: Applications to developmental research. Child Development.

[CIT0045] Van IJzendoorn M.H, Bakermans-Kranenburg M.J, Sagi-Schwartz A (2003). Are children of Holocaust survivors less well-adapted? A meta-analytic investigation of secondary traumatization. Journal of Traumatic Stress.

[CIT0046] Wetzels R, Matzke D, Lee M.D, Rouder J.N, Iverson G.J, Wagenmakers E.-J (2011). Statistical evidence in experimental psychology: An empirical comparison using 855 t tests. Perspectives on Psychological Science.

